# A Hybrid Non-destructive Measuring Method of Three-dimensional Profile of Through Silicon Vias for Realization of Smart Devices

**DOI:** 10.1038/s41598-018-33728-w

**Published:** 2018-10-26

**Authors:** Heulbi Ahn, Jaeseok Bae, Jungjae Park, Jonghan Jin

**Affiliations:** 1 Department of Science of Measurement, Korea University of Science and Technology (UST), 217, Gajeong-ro, Yuseong-gu, Daejeon 34113 Republic of Korea; 2 Division of Physical Metrology, Korea Research Institute of Standards and Science (KRISS), 267, Gajeong-ro, Yuseong-gu, Daejeon 34113 Republic of Korea

## Abstract

Smart devices have been fabricated based on design concept of multiple layer structures which require through silicon vias to transfer electric signals between stacked layers. Because even a single defect leads to fail of the packaged devices, the dimensions of the through silicon vias are needed to be measured through whole sampling inspection process. For that, a novel hybrid optical probe working based on optical interferometry, confocal microscopy and optical microscopy was proposed and realized for enhancing inspection efficiency in this report. The optical microscope was utilized for coarsely monitoring the specimen in a large field of view, and the other methods of interferometry and confocal microscopy were used to measure dimensions of small features with high speed by eliminating time-consuming process of the vertical scanning. Owing to the importance of the reliability, the uncertainty evaluation of the proposed method was fulfilled, which offers a practical example for estimating the performance of inspection machines operating with numerous principles at semiconductor manufacturing sites. According to the measurement results, the mean values of the diameter and depth were 40.420 µm and 5.954 µm with the expanded uncertainty of 0.050 µm (*k* = 2) and 0.208 µm (*k* = 2), respectively.

## Introduction

**Figure 1:**
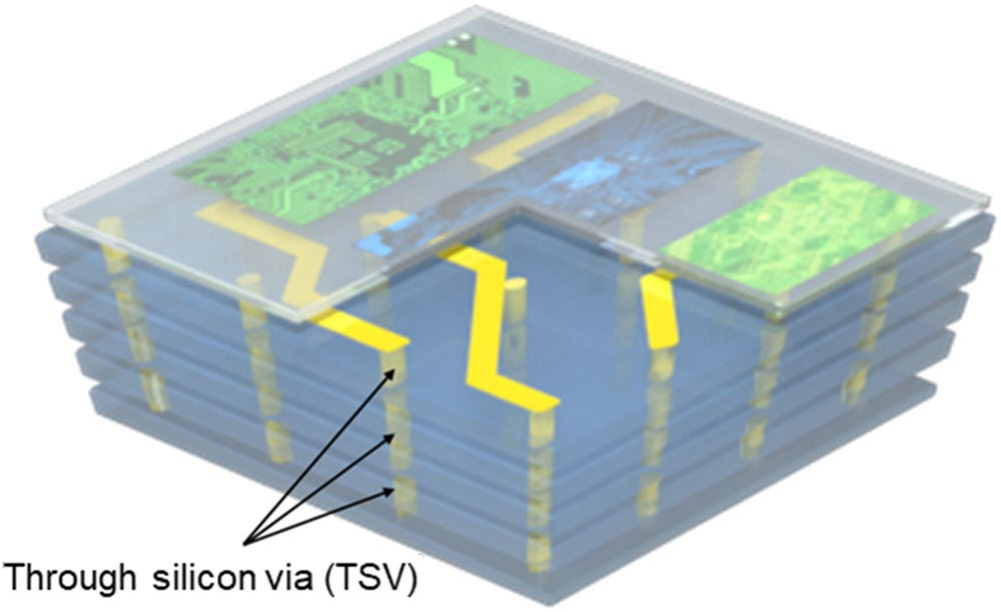
Schematic diagram of a smart integrated electric device. TSVs are required to be used for the electrical connection between the stacked layers, which are narrow and deep holes filled with conductive materials.

In recent years, smart devices with good portability have become widely used. As a basic component of these products, semiconductor circuits are being designed and developed in the form of miniaturized chips with various functions. To realize this, several single-layered electric circuits fabricated by existing lithography techniques are vertically stacked so that three-dimensional semiconductor packages can be created. Through silicon vias (TSVs) are required to be used for the electrical connection between the stacked layers as an indispensable component for stacked circuits, which are narrow and deep holes filled with conductive materials, as shown in Fig. 1. Depending on the purpose of use, these TSVs are designed and fabricated in various forms with different diameters and depths. In order to reduce the defect rate and improve the throughput of the stacked circuits, it is necessary to inspect whether the diameter and depth of the TSVs were fabricated with desired values. For instance, the shortened depth of the TSV leads to a disconnection of the electric signals transferring between stacked layers. Moreover, an irregular diameter of the TSV may cause defects such as voids and overhangs during the filling process of conductive materials^1^. Because an integrated circuit can fail even if only a single defect exists after all manufacturing processes, manufacturers want to measure the dimensions of TSVs through the whole sampling inspection process. However, due to the small size and high aspect ratio of the holes, the use of scanning electron microscopy and transmission electron microscopy with resolutions of tens of nanometers has become common at industrial sites as convenient methods, despite the fact that the specimen will be damaged when it is cut to measure its cross-section^2–7^. The preparation of the sample for measurements requires a longer inspection time, so that scanning electron microscope and transmission electron microscope are utilized as offline instruments.

For the practical realization of whole sampling inspections, non-destructive measuring methods based on optical methods have been suggested and demonstrated based on diffraction theory, scattering theory, spectroscopic reflectometry, white-light scanning interferometry, and spectral-domain interferometry in some previous studies^8–34^. The measuring method using diffraction theory could not measure the surface profile of each TSV but inspect only uniformity of the TSV array as macro inspection within a certain area^24,25^. Diameter and depth measurement methods based on scattering theory have also been proposed and realized^26^. A theoretical model was constructed with information about the refractive index of the substrate and the design dimensions of the TSV. The theoretical model numerically created scattering patterns, which varied according to the input parameters of the diameter and the depth. We can find a created pattern, of which the error is minimal with the measured one. At that time, the input parameters can be the diameter and depth of the TSV as optimal solutions. Even if this method never required any mechanical scanning, some information for constructing the theoretical model should be known in advance and the probing position should be precisely controlled to include an entire image of the TSV in the field of view. Similarly, spectroscopic reflectometers work on a theoretical model based on reflection^29,30^. They also need information about the refractive index of the substrate and the nominal dimensions of the TSV. On the other hand, to measure only the depth of the TSV, several methods based on spectral-domain interferometers have been proposed and demonstrated^31,32^. Owing to the low signal-to-noise ratio of the interference signals of the high-aspect ratio holes, the light was incident on the back side of the substrates. In this case, because the depth was calculated from the optical thickness, accurate information about the refractive index of the substrate was more important in this approach. Inaccurate information about the refractive index of the substrate causes major error in these types of measurements.

In this report, an all-in-one optical probe system is proposed and realized for measuring the dimensions of TSVs with a high aspect ratio. The three well-established metrological techniques of spectral-domain interferometry, confocal microscopy, and optical microscopy were merged into a single probe system for scientific and industrial use. Similarly, in other fields including medical areas and micro-device fabrication areas, such attempt to combine optical coherent tomography and confocal microscopy have been realized to investigate surface profiles or the tomography of various samples effectively^35–47^. Among three different techniques of the proposed method, optical microscopy is used to inspect or monitor small patterns coarsely to improve the efficiency of the inspection. If certain aspects to be investigated more precisely are found, the spectral-domain interferometer and the confocal microscope are utilized for accurate measurements of the depth and the diameter, respectively. Using the polarization property, another technical discrimination in this work, simultaneous measurements of the spectral-domain interferometer and the confocal microscope are facilitated by separating the lights reflected from the specimen and the reference mirror. By combining these techniques complementarily, the proposed method eliminates the need for the most time-consuming process of vertical scanning, which is essential for conventional optical interferometers and confocal microscopes while maintaining their individual advantages. Therefore, the proposed system contributes to reducing the inspection time and enhancing the detection rate of fine defects. It can also be used to measure the dimensions of other small patterns, including TSVs.

In terms of the reliability of the manufacturing process, the proposed method deals with the measurement reliability based on Guide to the expression of Uncertainty in Measurement (GUM) and on ISO guidelines (ISO 5436-1, ISO 5725-1, and ISO 5725-2)^48–51^. At research laboratories and industrial sites, several different types of meteorological instruments, such as atomic force microscope, scanning electron microscope, transmission electron microscope and scanning probe microscope, are utilized complementarily. When measuring the same sample with different instruments, the measured values might not be exactly coincided each other. In this case, it is knotty to judge if the measured values are reasonable or not even though the values are distributed within a small range. Therefore, it is easy to investigate the reliability of the measured values by checking whether the other instruments indicate values being within the uncertainty range of the proposed method or not. Especially, most of the technologies based on image processing or stylus probe are practically difficult to provide their uncertainties in a strict way owing to the lack of a direct traceability chain or the difficulty in linking of the traceability chain to the length standard. In this point of view, the proposed method can offer an opportunity to verify or correct other metrological instruments by providing reference values and the uncertainty range.

## Method

**Figure 2:**
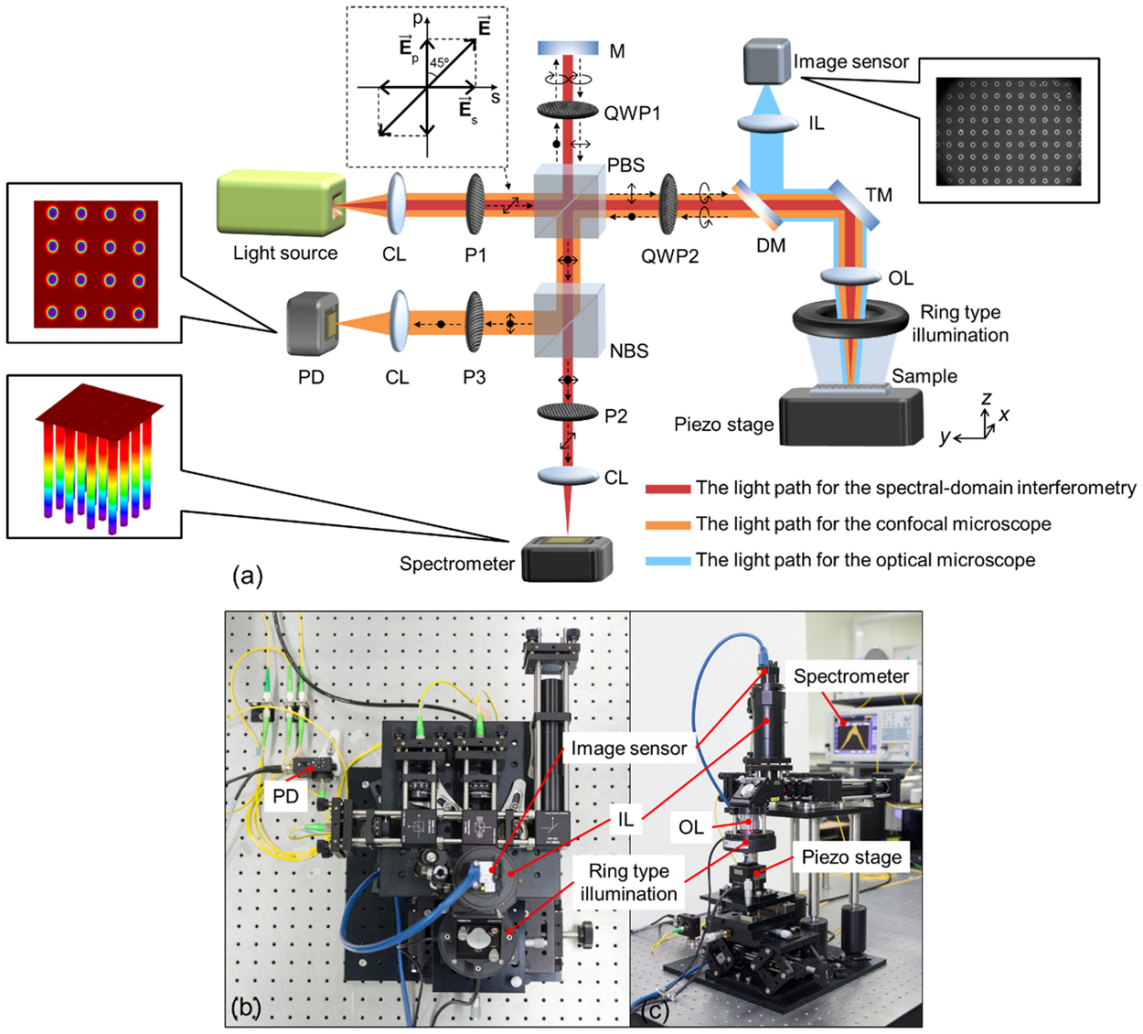
Optical layout and photographic view of the proposed method: (**a**) optical layout of the proposed method (Details of the polarization state are shown in Fig. A.1 of Appendix-A.), (**b**) photographical top view, and (**c**) side view of the experimental setup. (CL: collimation lens, P1, P2 and P3: polarizers, PBS: polarizing beam splitter, NBS: non-polarizing beam splitter, QWP1 and QWP2: quarter-wave plates, M: mirror, DM: dichroic mirror, TM: turning mirror, IL: imaging lens, OL: objective lens, PD: photo-detector, $\overrightarrow{{\bf{E}}}$: resultant electric field vector, ${\overrightarrow{{\bf{E}}}}_{{\rm{s}}}$: s component of the electric field vector, ${\overrightarrow{{\bf{E}}}}_{{\rm{p}}}$: p component of the electric field vector).

The proposed method operates in three different measuring modes: optical microscopy, confocal microscopy, and spectral-domain interferometry. Among these techniques, to share optical components and coincide with the measuring points of the confocal microscope and the spectral-domain interferometer, the polarization property of light was utilized. Figure 2(a) shows a schematic diagram of the proposed method with the polarization states. The light source has a spectral bandwidth of 50 nm (FWHM) at a center wavelength of 1550 nm in the near-infrared range. The polarizer (P1) was adjusted to create two orthogonal polarized lights on the s- and p-axes (See details in Appendix-A)^52^. Light emitted from the light source was linearly polarized at 45° to the vertical axis (p-axis) by passing it through the P1, as shown in Fig. 2(a). The resultant polarization of the light is the vector sum of two perpendicular polarizations on the s- and p-axes (${\overrightarrow{{\rm{E}}}}_{{\rm{s}}}$ and ${\overrightarrow{{\rm{E}}}}_{{\rm{p}}}$). The light was split using the polarizing beam splitter (PBS) into two orthogonal linearly polarized lights on the s- and p-axes. The vertically polarized light reflected from the PBS becomes circularly polarized through the quarter-wave plate (QWP1), of which the fast axis was located at 45° relative to the p-axis. The light was directed to the mirror (M). After being reflected at the mirror, the circularly polarized light passed through the QWP1 again and became horizontally polarized. The light went through the PBS and was directed to the non-polarizing beam splitter (NBS) with a 50:50 split ratio. Similarly, the horizontally polarized light transmitted through the PBS was directed to the quarter-wave plate (QWP2) whose fast axis was aligned at 45° relative to the p-axis, by which the light was circularly polarized after being transmitted. This light was delivered to the sample perpendicularly by the turning mirror (TM) and then reflected. After passing through QWP2 again, the circularly polarized light was converted into vertically polarized light. The vertically polarized light is reflected at the PBS and then directed to the NBS. The two orthogonal lights reflected from the M and the sample, respectively, were split by the NBS and traveled to the spectrometer and the photo-detector (PD). To create the interference spectrum between two orthogonal polarization components at the spectrometer, the transmission angle of polarizer (P2) was adjusted to 45° with respect to the vertical axis, which served as an analyzer. The combined lights reflected from the M and the sample can be simply expressed by Eq. (1) in terms of the speed of light in a vacuum (*c*), the optical frequency (*f*), and the optical path difference (*L*). The interference signal was captured by the spectrometer in the form of a spectrum in the wavelength domain. After converting and resampling the spectra into the frequency domain, the height value of the sample was easily calculated by analyzing the spatial period of the interference patterns. On the other hand, only light reflected from the sample was detected at the PD with the help of polarizer (P3), whose transmission axis was adjusted to 0° with respect to the vertical axis. If the surface was placed within the depth of focus, the intensity of the reflected light was high. Otherwise, the intensity of the reflected light became lower. The dimensions of the patterns can be determined more precisely from statistical analysis of the intensity map obtained by scanning the sample on the *x*-*y* plane with a two-dimensional translational piezo stage.

1 $$I(f,L)={I}_{0}(1+\,\cos (\frac{2\pi }{c}L\cdot f))$$

In addition, to examine the sample patterns and measurement locations, an imaging system was integrated onto the measurement system operating in the two modes of optical interferometry and confocal microscopy. For the imaging system, an objective lens having a magnification level of 50, a focal length of 120 mm and an image sensor 4.8 mm × 3.6 mm in size were adopted. The visible light for the microscopic images is delivered into the image sensor by reflection from the dichroic mirror (DM). In this report, for a feasibility test, a TSV having a nominal diameter of 5 µm and a nominal depth of 40 µm, a challenging component in a semiconductor foundry, was chosen as a sample.

## Results and Discussion

### Depth

**Figure 3:**
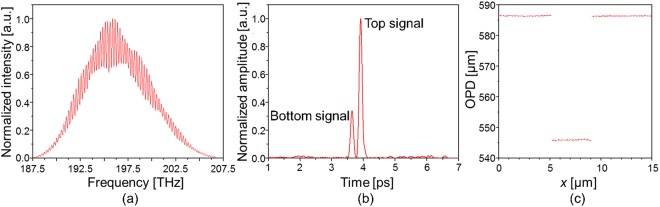
Interference spectrum and amplitude of its Fourier transform: (**a**) interference spectrum obtained at an edge of the TSV, (**b**) peaks representing for top surface and bottom surface of the TSV in the Fourier domain, and (**c**) depth profile of the TSV.

The 2048 points of the interference spectrum were sampled with an equal spacing in the wavelength range of 1450 nm to 1600 nm. The spectrum in the wavelength domain is converted to that in the frequency domain, after which it is interpolated to have the same sampling step of 9 GHz (=19.362 THz/2048) in the frequency domain, as shown in Fig. 3(a). The converted spectrum in the frequency domain was Fourier-transformed with 2^10^ zeros to detect the locations of the peaks, as shown in Fig. 3(b)^53^. As an example, Fig. 3(b) shows two peaks representing the top surface and the bottom surface at the edge of the TSV. According to the measurement positions, a single peak or both peaks can be observed. To plot the depth profile, only the strong peak is selected when dual peaks are observed. While the sample is scanned in the range of 15 µm along the *x*-axis, the surface profile was obtained as shown in Fig. 3(c). The depth of the profile can be analytically determined as a represented value using the ISO guideline of ISO 5436-1^49^. After 30 repeated measurements of the surface profile, the mean value and standard deviation of the depth of the TSV were 40.420 µm and 0.023 µm, respectively.

The uncertainty for the depth is affected by two major uncertainty components of the measurement repeatability (u_rep_(d)) and the analysis algorithm based on the international standard guideline of ISO 5436-1 (u_ISO_(d))^49^. First, the uncertainty with regard to the repeatability of the measurement is easily evaluated to be 4 nm after 30 consecutive measurements, which was the standard error of the mean value of 40.420 µm. Secondly, u_ISO_(d) is estimated based on a Monte Carlo simulation with consideration of the uncertainty of the optical path differences (u(OPD)) at multiple optical path difference (OPD) values on the depth profile. The uncertainty of the OPD is composed of the uncertainties of the discrete Fourier transform (DFT), the spectrometer, refractive index of air, and the long-term stability. The uncertainty of the discrete Fourier transform (u_DFT_(OPD)) is estimated using the ideally created spectra with identical measurement conditions of the wavelength range, number of data instances, the spectrum distribution, and the OPD. The relative error of the DFT algorithm in use is less than 4.5 × 10^−5^ within the OPD range of 500 µm to 600 µm. The uncertainty of the spectrometer (u_sp_(OPD)) is provided by the manufacturer, which is 0.01 nm in terms of the wavelength. In our laboratory, the uncertainty of the refractive index of air (u_air_(OPD)) is estimated to be less than 10^−6^, which can be disregarded owing to the small OPD of less than 1 mm. The uncertainty of the long-term stability (u_st_(OPD)) is estimated by measuring the OPDs every one minute for eight hours from 10 am to 6 pm. The standard deviation of the 480 OPDs is 44 nm. Therefore, u(OPD) was calculated to be 47 nm at an average OPD of 566 µm.

To estimate the uncertainty in the analysis algorithm of the depth, 10,000 sets of OPD values for the depth are numerically created by randomly adding or subtracting the uncertainty of the OPD with a normal distribution (See details in Appendix-B.1). The variance of the normal distribution equals the square of the uncertainty for the OPD, (47 nm)^2^. By analyzing the created OPD values, the uncertainty of the analysis algorithm is evaluated to be 25 nm. The combined uncertainty for the depth is the square root of the squared sums of all of the uncertainties of the measurement repeatability and the analysis algorithm. Finally, the expanded uncertainty of the depth is found to be 50 nm (*k* = 2). Table 1 summarizes the uncertainty components for determining the depth.

**Table 1:** Uncertainty budget of the depth measurement.

Source of uncertainty	Sensitivity coefficient	Uncertainty value @ OPD = 566 μm
Uncertainty of the measurement repeatability of the depth, u_rep_(d)		4 nm
Uncertainty of the analysis algorithm, u_ISO_(d)		25 nm
Uncertainty of determining the optical path differences, u(OPD)		47 nm
Uncertainty of the discrete Fourier transform, u_DFT_(OPD)	2.62 × 10^−5^	
Uncertainty of the spectrometer, u_sp_(OPD)	6.6 × 10^−6^	
Uncertainty of the refractive index of air, u_air_(OPD)	10^−6^	<1 nm
Uncertainty of the long-term stability, u_st_(OPD)		44 nm
Expanded uncertainty of the depth, U(d)		50 nm (*k* = 2)

### Diameter

**Figure 4:**
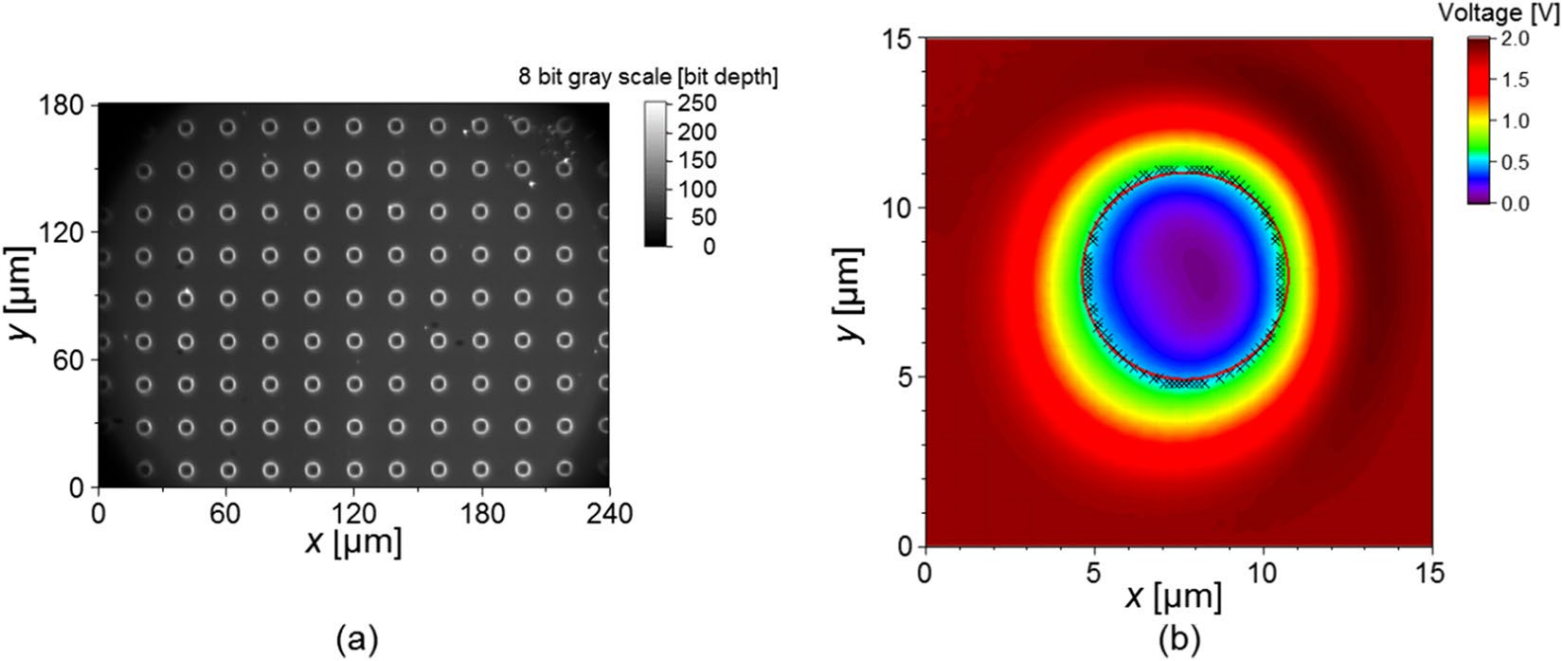
Measurement results: (**a**) microscopic image of the TSV, and (**b**) circular fitting of edge points of the TSV on the intensity map obtained by the confocal microscope.

For a TSV located at the center of the sample in Fig. 4(a), a two-dimensional intensity map is obtained in the confocal microscope mode, as shown in Fig. 4(b). To distinguish between the top surface and the bottom surface, edge points are defined as a set of points having a pre-determined threshold, the value of which was 30% of the mean intensity obtained at the top surface. The selected edge points are circularly fitted to estimate the diameter using least square method. According to the result of 30 repeated measurements, the mean value of the diameter was 6.091 µm with a standard deviation of 0.009 µm. For reliable measurements, the diameter value was calibrated with a correction value of −0.137 µm, which is an offset between the mean diameters determined by the proposed method and a toolmaker’s microscope as a standard instrument being traceable to the length standard^54^ (See details in Appendix-B.2).

The uncertainty when determining the diameter is caused by two major uncertainty components, which are the measurement reproducibility and the calibration process^50,51^. According to ISO 5725-1 and ISO 5725-2, we attempted to estimate the reproducibility of diameter measurements considering different factors of the environment, the time which elapsed between the measurements, different locations of the *x*-*y* PZT stage, and different orientations of the sample^50,51^. The uncertainty of the measurement reproducibility (u_Rep_(*ϕ*)) is estimated to be only 13 nm, which is the standard deviation of 40 sets of diameter values analyzed by circularly fitting the edge points of the intensity map. The uncertainty of the calibration process (u_cal_(*ϕ*)) come directly from the combined uncertainty of the standard instrument, which is found to be 0.103 µm (*k* = 1) with an average value of 5.954 µm. Therefore, the expanded uncertainty of the diameter is 0.208 µm (*k* = 2). Table 2 summarizes the uncertainty components used to determine the diameter.

**Table 2:** Uncertainty budget of the diameter measurement.

Source of uncertainty	Uncertainty value @ *ϕ* = 5.954 μm
Uncertainty of the measurement reproducibility of the diameter, u_Rep_(*ϕ*)	13 nm
Uncertainty of the calibration process, u_cal_(*ϕ*)	103 nm
Expanded uncertainty of the diameter, U(*ϕ*)	208 nm (*k* = 2)

### Integration work

**Figure 5:**
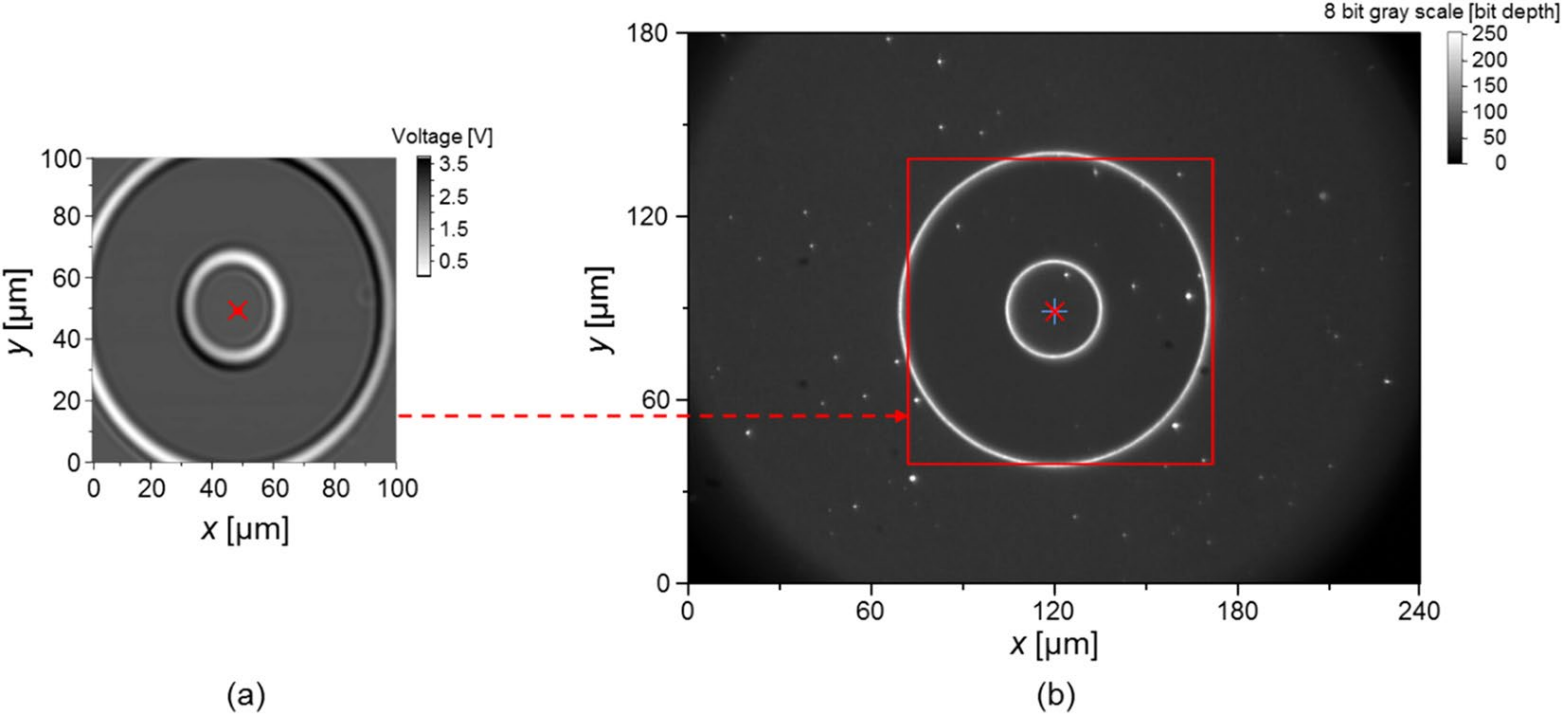
Determination of the measuring point of the hybrid method: (**a**) Confocal microscopic image of the circular pattern in a step-height-certified reference material obtained by scanning the sample, and (**b**) Optical microscopic image of the circular pattern in a step-height-certified reference material. (The ‘×’ markers in (**a** and **b**) represent the center points of the circular pattern. The ‘+’ maker and rectangular box in (**b**) are the center pixel of the image sensor and the scanned area of the confocal microscope shown in (**a**) respectively).

**Figure 6:**
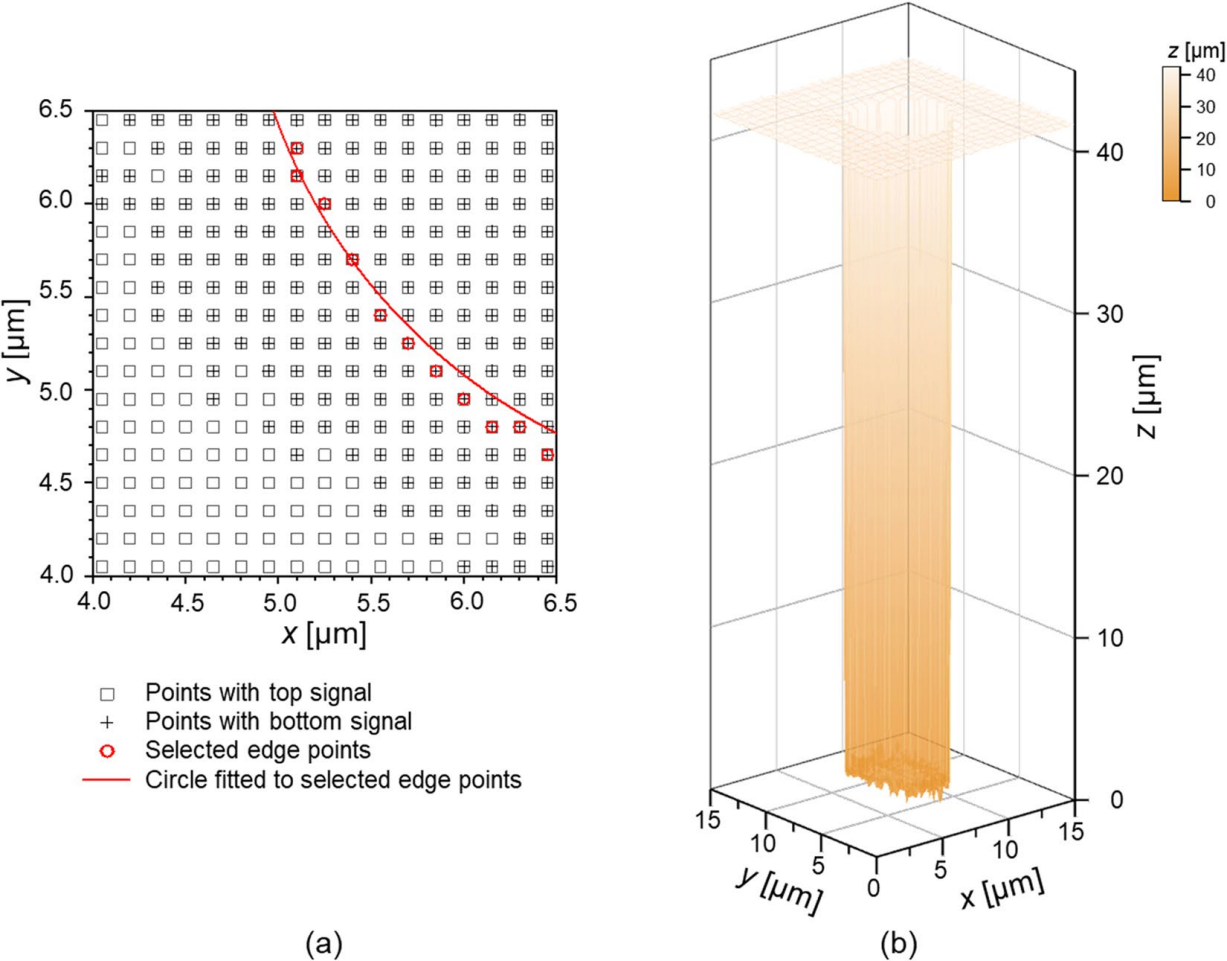
Measurement results: (**a**) the indexed map representing the appearance of top or bottom signal near the edge of the TSV with the fitted circle, and (**b**) three-dimensional surface profile of the TSV (The surface profile was obtained by combining the intensity map in Fig. 4 (**b**) with the OPD map in (**a**). Inside of the edges in (**a**), OPDs corresponding to the bottom surface were selected. Otherwise, OPDs corresponding to the top surface were selected outside of the edges).

There are two tasks to integrate optical microscopy, confocal microscopy, and spectral domain interferometry. One is to define where the measuring points of both the confocal microscope and the spectral-domain interferometer are located in the microscopic image. The other is to combine the measured data obtained by the spectral-domain interferometer with that obtained by the confocal microscope to create 3D profile of the TSV. First, for proper working of the integrated system, measuring points for single-point measuring methods of the confocal microscope and spectral-domain interferometer should be defined exactly within the microscopic image. To do this, especially along the *x*- and *y*-axes, circular patterns with diameters of 30 µm and 100 µm fabricated on a step-height-certified reference material (SH001M-0002, KRISS) are measured using the confocal microscope, as shown in Fig. 5(a). At the center point of the circular pattern in the intensity map, as denoted by the ‘×’ marker in Fig. 5(a), the microscopic image was obtained in a field of view of 240 µm × 180 µm, as shown in Fig. 5(b). The location of the image sensor was carefully adjusted so that a center point of the circular pattern in the microscopic image coincides with the center pixel of the image sensor. Here, the point marked with ‘+’ and the rectangular box in Fig. 5(b) are the center pixel of the microscopic image and the scanned area of the confocal microscope, respectively. Because the confocal microscope and the spectral-domain interferometer share measurement points owing to sharing of the objective lens in use, the center pixel of the microscopic image can be regarded as the measuring point for both methods. Secondly, to create a 3D profile of the TSV, we use the intensity map and the OPD map obtained by the PD and the spectrometer, respectively. When plotting the OPD map, two OPDs might be observed at some locations (near the edge or the bottom surface of the TSV), where it has dual peaks in the Fourier-domain. Figure 6(a) shows indexed map representing the appearance of top (The ‘□’ marker) or bottom (The ‘+’ marker) signals near the edge of the TSV with the fitted circle. To select a proper OPD value from the dual OPDs at these locations (The ‘’ positions in Fig. 6(a)), the edges of the TSV (The red ‘○’ positions) were determined from the intensity map obtained from the confocal microscope. Inside the edges, OPDs corresponding to the bottom surface were selected. Otherwise, OPDs corresponding to the top surface were selected. Therefore, we can have the three-dimensional profile of the TSV, as shown in Fig. 6(b). As an example of an efficient inspection process, the patterns are roughly monitored and observed by examining microscopic images. If defects, particles or faults are found in the previous rough step, the interferometer and confocal microscope are utilized as precision tools.

## Summary

With the advent of smart devices, a new design concept has been introduced to build 3D stacked semiconductors. During the realization of this concept, numerous small structures such as TSVs are newly fabricated and widely utilized. To enhance the throughput of this new manufacturing process, a non-destructive metrological method was proposed as a hybrid system based on an optical microscope, a spectral-domain interferometer, and a confocal microscope. As a challenging sample, one of the finest TSVs in semiconductor foundries was selected. The optical microscope provided microscopic images of the top view of the TSV to inspect defects roughly in a wide field of view. As precision tools offering nanometer resolutions, the spectral-domain interferometer and the confocal microscope measured the depth and the diameter values, respectively. By sharing a measuring point of each technique, the hybrid measuring system could work efficiently. According to the measurement results, the mean values of the depth and the diameter of the TSV were respectively 40.420 µm and 5.954 µm with corresponding standard deviations of 0.023 µm and 0.013 µm. For reliable measurements, the uncertainty evaluations on the depth and diameter were fulfilled based on the GUM, ISO 5436-1, ISO 5725-1 and ISO 5725-2^48–51^. According to the uncertainty budget, the expanded uncertainty for measuring the depth was 0.050 µm (*k* = 2), consisting of standard uncertainties related to measurement repeatability and the analysis algorithm based on the international standard guideline of ISO 5436-1^49^. In addition, the expanded uncertainty when measuring the diameter was 0.208 µm (*k* = 2), stemming from the standard uncertainties of the measurement reproducibility and the calibration process^50,51^.

## Electronic supplementary material

Appendix
