# Supplementary material for: A Hybrid Non-destructive Measuring Method of Three-dimensional Profile of Through Silicon Vias for Realization of Smart Devices
Source: Sci Rep. 2018 Oct 26;8:15342. doi: 10.1038/s41598-018-33728-w (PMC6203746; doi:10.1038/s41598-018-33728-w)
Supplement: Supplementary file 1 — Appendix [file 41598_2018_33728_MOESM1_ESM.docx]

**A Hybrid Non-destructive Measuring Method of Three-dimensional Profile of Through Silicon Vias for Realization of Smart Devices**

Heulbi Ahn^1^, Jaeseok Bae^1^, Jungjae Park^1,2^ & Jonghan Jin^1,2^

^1^Department of Science of Measurement, Korea University of Science and Technology (UST), 217, Gajeong-ro, Yuseong-gu, Daejeon, 34113, Republic of Korea. ^2^Division of Physical Metrology, Korea Research Institute of Standards and Science (KRISS), 267, Gajeong-ro, Yuseong-gu, Daejeon, 34113, Republic of Korea. Correspondence and requests for materials should be addressed to J. J. (email: jonghan@kriss.re.kr)

**Appendix-A. Polarization state**

This section explains the experimental setup and how the polarization is utilized in our setup. Fig. A.1. presents a simplified diagram and the light path for the spectral interferometry used in our setup.


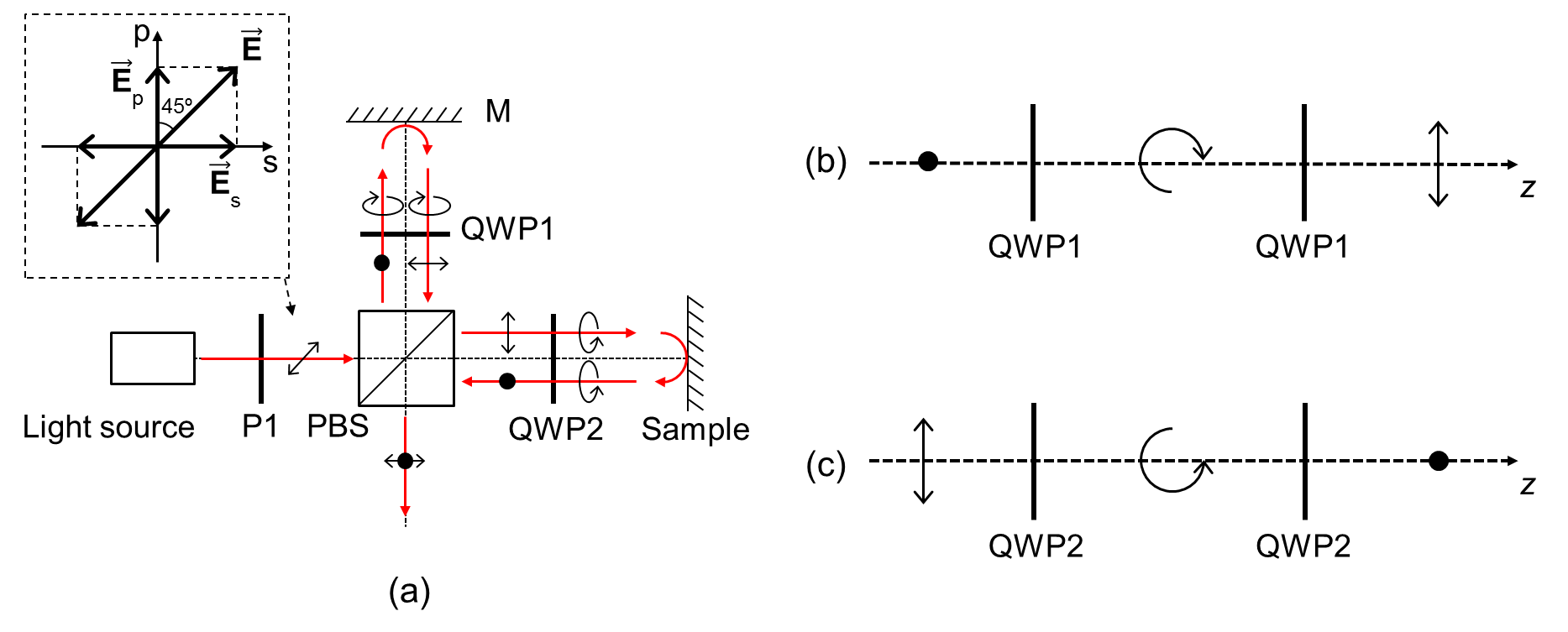


**Fig. A.1.** Schematic diagram of polarization state: (a) the proposed method. (b) Polarization state after passing through QWP1 which is the mirror path in Fig. A.1(a), and (c) Polarization state after passing through QWP2 which is the sample path in Fig. A.1(a) (M: mirror, QWP1 and QWP2: quarter wave plates, P1: polarizer, PBS: polarizing beam splitter, $\vec{\text{E}}$: resultant electric field vector, $\vec{\text{E}}_{\text{s}}$: s component of electric field vector, $\vec{\text{E}}_{\text{p}}$: p component of electric field vector)

The light propagating along the *z*-axis can be easily expressed by Eq. (A.1) in terms of the amplitudes of the s- and p-axes (*E*_0s_ and *E*_0p_), the wave number (*k*), the angular velocity (*ω*), and the phase offset between two orthogonally polarized components (*ε*). The polarization can be written in Jones matrix form with the phase terms of *φ*_s_ and *φ*_p_, as indicated by Eq. (A.2)^52^.

(A.1)

$\overrightarrow{\mathbf{E}}=\left[\begin{matrix} E_{0\text{s}}e^{i\phi_{\text{s}}} \\ E_{0\text{p}}e^{i\phi_{\text{p}}} \end{matrix}\right]$ (A.2)

The light propagating from the light source to the spectrometer through the polarizer (P1) and a round trip of the polarization beam splitter (PBS), quarter wave plate (QWP1) and mirror (M) is written in Jones matrix form, as expressed by Eq. (A.3). Similarly, Eq. (A.4) represents the light propagating from the light source to the spectrometer through the P1 and a round trip of the PBS, QWP2 and the sample in Jones matrix form^52^.

(A.3)

(A.4)

where, **T***_QWP1_* and **T***_QWP2_* are Jones matrixes for the QWP1 and QWP2, respectively. $\vec{\text{E}}_{h}$ and $\vec{\text{E}}_{v}$ are horizontal polarization state and vertical polarization state, respectively. Amplitude coefficients are ignored for convenience.

**Appendix-B. Evaluation of the measurement uncertainty**

**B.1. Uncertainty of the depth measurement**

To estimate the uncertainty in the analysis algorithm for the depth, 10,000 sets of OPD values for the depth are numerically created by randomly adding or subtracting the uncertainty of the OPD with a normal distribution. The variance of the normal distribution equals the square of the uncertainty for the OPD, (47 nm)^2^. By analyzing the created OPD values, the uncertainty of the analysis algorithm is evaluated to be 25 nm. For example, in the ideal case shown in Fig. B.1(a), the uncertainty of the OPD equals 0 nm. This results in an identical depth of the TSV at all times (i.e., the uncertainty of the depth is also zero). Because the depth was determined by analyzing 16 OPD values, we should consider the uncertainty of the OPD when creating OPD #1 to OPD #16 independently, as shown in Fig. B.1(b).


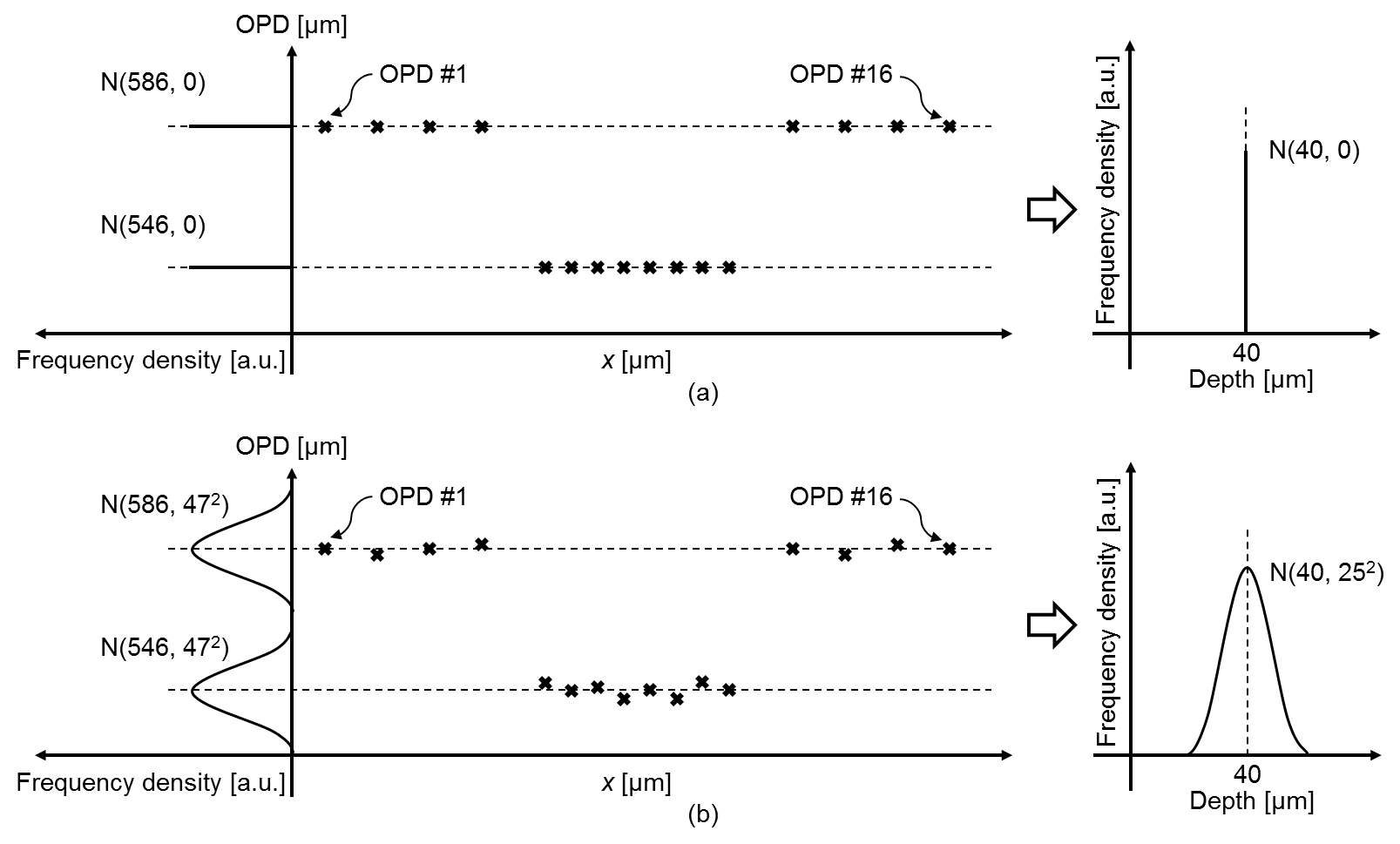


**Figure B.1.** Surface profile with the frequency density of the OPDs of the surface profile and the distribution of the depth: (a) An ideal case of the OPDs of the surface profile, and (b) An example case of the OPD values created for the Monte Carlo simulation

The combined uncertainty for the depth is the square root of the squared sums of all of the uncertainties of the measurement repeatability and the analysis algorithm, as expressed in Eq. (B.1). Finally, the expanded uncertainty of the depth is found to be 50 nm (*k* = 2). Table 1 summarizes the uncertainty components for determining the depth. Figure B.2 shows a schematic diagram of the uncertainty propagation of the depth of the TSV.

$\text{u(d) }=\text{[u}_{\text{rep}}^{2}\text{(d)}+\text{u}_{\text{ISO}}^{2}\text{(d)]}^{\text{1/2}}$ (B.1)


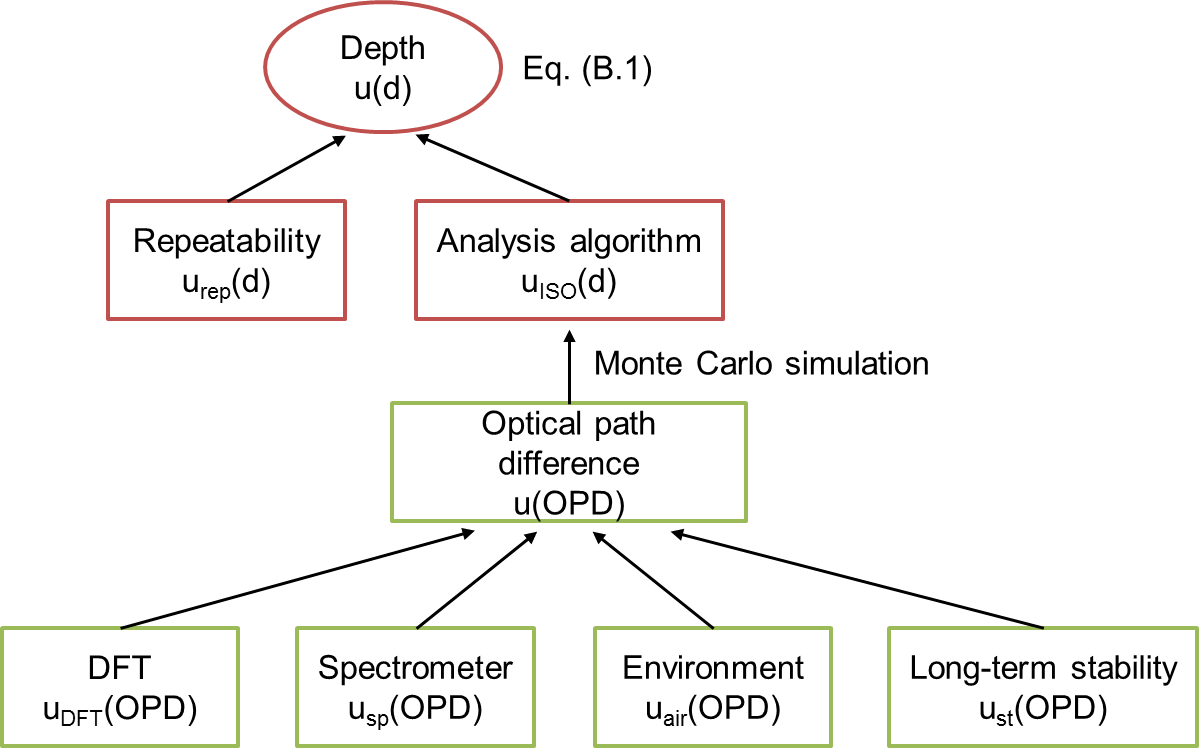


**Figure B.2.** Schematic diagram of the uncertainty propagation of the depth of the TSV. The uncertainty for the depth was affected by two major uncertainty components of the measurement repeatability and the analysis algorithm based on the international standard guideline of ISO 5436-1. The uncertainty for the analysis algorithm of the depth was estimated based on a Monte Carlo simulation with consideration of the uncertainty of the optical path differences, which was composed of the uncertainties of the discrete Fourier transform (DFT), the spectrometer, refractive index of air, and the long-term stability.

**B.2. Uncertainty of the diameter measurement**

B.2.1 The comparator calibration procedure^54^

The comparator used for the diameter comparison is a toolmaker’s microscope developed by the Korea Research Institute of Standards and Science to disseminate line standards, such as the linewidth and line spacing, which are utilized for calibration service. There are two tasks to be done, magnification calibration and threshold level calibration, when calibrating the comparator. The first task is to determine the actual length corresponding to a single pixel upon a microscope image for each magnification when using different objective lenses, referred to as magnification calibration. As the first step of this calibration, two calibration gratings with nominal pitches of 3 μm (TGG01, 1-D array of triangular steps, MikorMasch) and 10 μm (TGZ11, 1-D arrays of rectangular SiO2 steps on a Si wafer, MikorMasch) are measured by a metrological atomic force microscope developed by the Korea Research Institute of Standards and Science, resulting in mean pitches of 2999.9 nm and 9999.4 nm, respectively. In the second step, the same specimens are measured by the toolmaker’s microscope. Specifically, the numbers of pixels corresponding to the pitches in nine different positions equally distributed by a 3 by 3 array in the center part of each specimen are measured and averaged to each representative value for five different objective lenses with magnification levels of 5, 10, 20, 50, and 100. After these two steps, magnification calibration is accomplished by taking the measured pitch value divided by the number of pixels measured at each magnification, as shown below.

(unit: μm/pixel)

| x5 | x10 | x20 | x50 | x100 |
| --- | --- | --- | --- | --- |
| 0.6796 | 0.3384 | 0.1707 | 0.06752 | 0.03386 |

**Table B.1.** Calibrated magnification of each objective lens of microscope^54^

The second task is to determine a proper threshold level upon the intensity profile of the linewidth pattern measured by the toolmaker’s microscope for the linewidth measurement. The linewidth is determined by the distance between two intersection points of the measured intensity profile and the threshold level. For this task, images of linewidth patterns of a standard reference material (SRM475, chrome patterns on a quartz plate, National Institute of Standards and Technology) having various linewidth patterns with a linewidth range from 1 μm to 10 μm are obtained from the toolmaker’s microscope. Specifically, for a certain linewidth pattern, an intensity profile is measured, after which a threshold level which gives the linewidth measured by the threshold level a value similar to the certified linewidth value provided by National Institute of Standards and Technology is determined. This procedure was repeated here for nine different linewidth patterns, resulting in a representative value averaged over nine threshold levels.

B.2.2 The uncertainty evaluation procedure of linewidth measurement using toolmaker’s microscope^54^

the uncertainty of the linewidth measurement using the toolmaker’s microscope was calculated by combining three types of standard uncertainties. The first of these is the standard uncertainty (u(*P*_0_)) of the certified value of the standard specimen used for magnification calibration of the toolmaker’s microscope, which was calculated to be 1.95 nm by dividing the expanded uncertainty (3.9 nm) provided in the certificate by 2. The second is the standard uncertainty (u(*P*'_0_)) of the measured value of the magnification calibration specimen, in pixels. With regard to u(*P*'_0_), there are two different standard uncertainties related to the non-uniformity (u(*P*'0_*Uni*)) and the measurement repeatability (u(*P*'0_*Rep*)) of the magnification calibration specimen. The standard deviation of the pitch values measured at five different positions upon the magnification calibration specimen were calculated to be 0.66 in pixels, which resulted in a u(*P*'0_*Uni*) value of 0.295 in pixels by dividing 0.66 by √5. The u(*P*'0_*Rep*) value was evaluated to be 0.295 in pixels by taking the pooled experimental standard deviation of the pitch values measured 50 times over one year. The calculated value of u(*P*'_0_) was 0.417 in pixels, as determined by combining u(*P*'0_*Uni*) and u(*P*'0_*Rep*). The third type of uncertainty is the standard uncertainty (u(*L*')) of the measured value of the linewidth standard specimen, in pixels. For u(*L*'), there are four different standard uncertainties related to the resolution limit (u(*L*'*_Res_*)) of the CCD camera, the threshold level determination for the detection of edges (u(*L*'*_Edg_*)), the non-uniformity (u(*L*'*_Uni_*)), and the measurement repeatability (u(*L*'*_Rep_*)) of the linewidth standard specimen. Because the resolution for determining the edge position can typically be enhanced to less than 0.1 in pixels through a mathematical calculation, u(*L*'*_Res_*) was calculated to be 0.029 in pixels by dividing 0.1 by 2√3 upon consideration of the rectangular probability distribution. The value of u(*L*'*_Edg_*) was estimated from the variation of the measured linewidth value when the threshold level is changed by approximately 5 %, which resulted in a value of 3.788 in pixels. As a result, u(*L*'*_Edg_*) was calculated to be 2.187 in pixels by dividing 3.788 by √3 considering the rectangular probability distribution. The standard deviation of the linewidth values measured at five different positions upon the linewidth standard specimen were calculated to be 1.98 in pixels, which resulted in a value of u(*L*'*_Uni_*) of 0.886 in pixels after dividing 1.98 by √5. Lastly, u(*L*'*_Rep_*) was estimated from the standard deviation of the linewidth values measured five times, which resulted in a value of 0.59 in pixels by dividing the standard deviation of 1.32 in pixels by √5. As a result, u(*L*') was calculated to be 2.432 in pixels by combining u(*L*'*_Res_*), u(*L*'*_Edg_*), u(*L*'*_Uni_*), and u(*L*'*_Rep_*). After the calculation of all three standard uncertainties of u(*P*_0_), u(*P*'_0_), and u(*L*'), the combined standard uncertainty (u*_c_*(*L*)) was calculated as shown in Eq. (B.2).

(B.2)

where *L* means the measured value of the linewidth specimen in μm, *P*_0_ means the certified value of the magnification calibration specimen in μm, *P*'_0_ means the measured value of the magnification calibration specimen in pixels, and *L*' means the measured value of the linewidth standard specimen in pixels. Because the diameter of the TSV was measured by the toolmaker’s microscope for a diameter comparison in our work, the mean value of the TSV diameter measured over 30 times, which is 5.954 μm, was used as the value of *L*. With *P*_0_ equal to 2999.9 nm, *P*'_0_ set to 88.597 in pixels, and *L*' equal to 146.19 in pixels, u*_c_*(*L*) was calculated and found to be about 103 nm (*k* = 1). The following table shows a summary of the uncertainty budget.

| Uncertainty components | Probability distribution | Standard uncertainty | Sensitivity coefficients |
| --- | --- | --- | --- |
| u(*P*_0_) | Normal | 1.95 × 10^-3^ μm | 1.98 |
| u(*P*'_0_) | Normal | 0.417 pixel | 0.0672 μm/pixel |
| u(*P*'0_*Uni*) | *t* | 0.295 pixel | 1 |
| u(*P*'0_*Rep*) | Normal | 0.295 pixel | 1 |
| u(*L*') | Normal | 2.432 pixel | 0.0407 μm/pixel |
| u(*L*'*_Res_*) | Rectangular | 0.029 pixel | 1 |
| u(*L*'*_Edg_*) | Rectangular | 2.187 pixel | 1 |
| u(*L*'*_Uni_*) | *t* | 0.886 pixel | 1 |
| u(*L*'*_Rep_*) | Normal | 0.59 pixel | 1 |
| u*_c_*(*L*) | Normal | 0.103 μm |  |

**Table B.2.** Uncertainty budget of the linewidth measurement

The value of u*_c_*(*L*) originating from the comparator was reflected in the uncertainty budget of the diameter measurement as a type of uncertainty in the calibration process (u_cal_(*ϕ*)) in our work.

B.2.3 The uncertainty evaluation procedure of the diameter measurement

The estimation of the reproducibility is carried out as follows. With a random time interval in 20 days, the total 40 sets of measurements were carried out. At each set, we measured the same TSV 30 times consecutively using the same procedure at different orientations, that is, the locations and angles were varied. During the entire measurement process, the environmental factors such as the temperature and relative humidity have also changed slightly. Table B.3 provides the measurement results and conditions. Because the precision is normally expressed in terms of the standard deviation, the standard deviation of 40 mean values of the diameter can be estimated as the ‘reproducibility’, which can include the ‘repeatability’ and ‘long-term stability’ as mentioned in ISO 5725-1 and ISO 5725-2^50, 51^. According to these results, the reproducibility was determined to be 13 nm. Hence, the uncertainty of the reproducibility (u_Rep_(*ϕ*)) was 13 nm. The uncertainty of the calibration process (u_cal_(*ϕ*)) comes directly from the combined uncertainty of the standard instrument, which was found to be 0.103 µm (*k* = 1) with an average value of 5.954 µm. Therefore, the expanded uncertainty of the diameter was expressed by Eq. (B.3), with the result being 0.208 µm (*k* = 2). Table 2 summarizes the uncertainty components used to determine the diameter. Figure B.3 shows a schematic diagram of uncertainty propagation of the diameter of the TSV.

$\text{u(}\varphi\text{) }=\text{[u}_{\text{Rep}}^{2}\text{(}\varphi\text{)}+\text{u}_{\text{cal}}^{2}\text{(}\varphi\text{)]}^{\text{1/2}}$ (B.3)


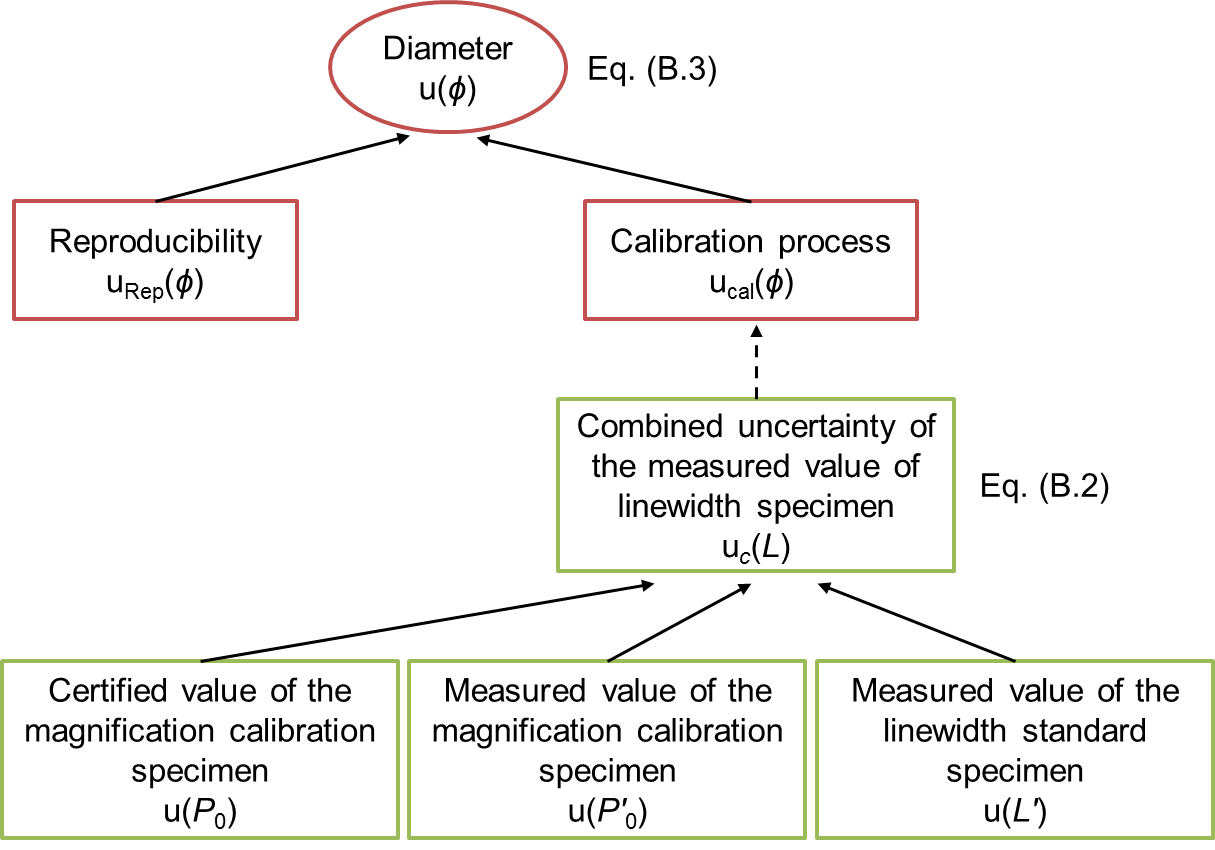


**Figure B.3.** Schematic diagram of the uncertainty propagation of the diameter of the TSV. The uncertainty when determining the diameter stemmed from two major uncertainty components: the measurement reproducibility and the calibration process. The uncertainty of the calibration process came directly from the combined uncertainty of the standard instrument in use.

| Number | Date and time | Environmental factors | | Different orientations | | Measurement results (Diameter) | |
| --- | --- | --- | --- | --- | --- | --- | --- |
|  |  | Temperature  (ºC) | Relative humidity  (% R.H.) | Location of the TSV  (*x* μm, *y* μm) | Angle of the sample  (º) | Mean value  (μm) | Standard deviation  (μm) |
| 1 | 1 August 2018, 23:29 | 23.5 | 41.5 | (33.0 , 13.5) | 0 | 6.065 | 0.009 |
| 2 | 4 August 2018, 04:39 | 23.6 | 41.2 | (14.0, 51.0) | -5 | 6.067 | 0.013 |
| 3 | 4 August 2018, 16:06 | 23.5 | 41.3 | (59.0, 28.0) | 64 | 6.077 | 0.009 |
| 4 | 5 August 2018, 05:00 | 23.6 | 41.1 | (27.5, 47.8) | 120 | 6.066 | 0.009 |
| 5 | 5 August 2018, 16:49 | 23.6 | 40.9 | (36.9, 43.5) | 121 | 6.106 | 0.006 |
| 6 | 6 August 2018, 02:34 | 23.4 | 41.4 | (23.1, 53.7) | 9 | 6.077 | 0.009 |
| 7 | 6 August 2018, 04:37 | 23.4 | 41.6 | (49.9, 54.2) | 17 | 6.077 | 0.011 |
| 8 | 6 August 2018, 13:40 | 23.5 | 41.5 | (41.2, 74.0) | 29 | 6.069 | 0.009 |
| 9 | 6 August 2018, 17:54 | 23.5 | 42.4 | (55.3, 83.0) | 52 | 6.087 | 0.012 |
| 10 | 6 August 2018, 22:41 | 23.5 | 41.5 | (57.5, 75.3) | 65 | 6.097 | 0.007 |
| 11 | 7 August 2018, 01:05 | 23.5 | 41.8 | (41.4, 26.0) | 83 | 6.106 | 0.006 |
| 12 | 7 August 2018, 10:35 | 23.4 | 41.6 | (51.8, 52.7) | 105 | 6.094 | 0.004 |
| 13 | 7 August 2018, 16:50 | 23.4 | 41.7 | (8.5, 37.5) | 40 | 6.079 | 0.007 |
| 14 | 7 August 2018, 22:44 | 23.4 | 42.0 | (65.7, 27.9) | 84 | 6.087 | 0.005 |
| 15 | 8 August 2018, 02:27 | 23.5 | 41.7 | (32.1, 72.7) | 35 | 6.092 | 0.008 |
| 16 | 8 August 2018, 09:40 | 23.4 | 41.6 | (47.1, 64.2) | 20 | 6.086 | 0.006 |
| 17 | 8 August 2018, 13:56 | 23.5 | 41.8 | (21.3, 38.9) | 6 | 6.079 | 0.009 |
| 18 | 8 August 2018, 21:38 | 23.4 | 40.9 | (3.7, 6.78) | -16 | 6.072 | 0.008 |
| 19 | 9 August 2018, 06:28 | 23.5 | 41.7 | (26.3, 49.5) | -21 | 6.064 | 0.008 |
| 20 | 9 August 2018, 18:44 | 23.3 | 41.7 | (44.5, 78.5) | -2 | 6.088 | 0.009 |
| 21 | 9 August 2018, 22:42 | 23.5 | 41.6 | (49.8, 49.0) | 12 | 6.080 | 0.007 |
| 22 | 10 August 2018, 08:21 | 23.4 | 41.8 | (42.5, 76.2) | -8 | 6.086 | 0.011 |
| 23 | 10 August 2018, 14:24 | 23.4 | 41.7 | (67.5, 42.5) | 77 | 6.101 | 0.008 |
| 24 | 10 August 2018, 21:35 | 23.5 | 42.7 | (41.1, 46.3) | 24 | 6.062 | 0.010 |
| 25 | 11 August 2018, 02:33 | 23.5 | 43.1 | (38.3, 44.0) | 1 | 6.102 | 0.009 |
| 26 | 11 August 2018, 16:05 | 23.5 | 43.4 | (34.0, 13.3) | -5 | 6.095 | 0.009 |
| 27 | 11 August 2018, 18:39 | 23.5 | 43.5 | (50.4, 51.4) | 30 | 6.072 | 0.009 |
| 28 | 11 August 2018, 23:56 | 23.4 | 43.5 | (44.5, 73.5) | 8 | 6.075 | 0.007 |
| 29 | 12 August 2018, 03:28 | 23.4 | 43.4 | (46.8, 45.8) | 25 | 6.081 | 0.007 |
| 30 | 12 August 2018, 17:08 | 23.5 | 43.5 | (37.5, 55.4) | -74 | 6.080 | 0.006 |
| 31 | 12 August 2018, 21:06 | 23.4 | 42.7 | (38.8, 24.6) | 10 | 6.072 | 0.011 |
| 32 | 13 August 2018, 01:25 | 23.3 | 41.6 | (40.3, 6.35) | 18 | 6.082 | 0.007 |
| 33 | 13 August 2018, 07:35 | 23.3 | 41.8 | (38.7, 23.9) | -1 | 6.097 | 0.011 |
| 34 | 13 August 2018, 15:32 | 23.3 | 42.3 | (23.9, 48.7) | -27 | 6.098 | 0.006 |
| 35 | 14 August 2018, 21:10 | 23.4 | 43.1 | (40.5, 48.8) | 0 | 6.072 | 0.008 |
| 36 | 15 August 2018, 00:01 | 23.4 | 43.5 | (42.8, 43.7) | 15 | 6.073 | 0.007 |
| 37 | 17 August 2018, 21:17 | 23.4 | 42.5 | (37.6, 58.2) | 2 | 6.062 | 0.010 |
| 38 | 18 August 2018, 16:32 | 23.5 | 41.5 | (45.7, 71.9) | 29 | 6.073 | 0.010 |
| 39 | 19 August 2018, 11:53 | 23.3 | 41.9 | (40.5, 52.6) | -20 | 6.095 | 0.009 |
| 40 | 20 August 2018, 08:54 | 23.4 | 41.3 | (33.5, 41.4) | 2 | 6.076 | 0.008 |

**Table B.3.** Diameter measurement result of the TSV
